# PDL-1 Blockade Prevents T Cell Exhaustion, Inhibits Autophagy, and Promotes Clearance of Leishmania donovani

**DOI:** 10.1128/IAI.00019-18

**Published:** 2018-05-22

**Authors:** Samar Habib, Abdeljabar El Andaloussi, Khaled Elmasry, Aya Handoussa, Manar Azab, Aliaa Elsawey, Ayman Al-Hendy, Nahed Ismail

**Affiliations:** a Department of Obstetrics and Gynecology, Medical College of Georgia (MCG), Augusta University, Augusta, Georgia, USA; b Department of Medical Parasitology, Faculty of Medicine, Mansoura University, Mansoura, Egypt; c Department of Cellular Biology and Anatomy, Medical College of Georgia, Augusta University, Augusta, Georgia, USA; d Department of Human Anatomy & Embryology, Faculty of Medicine, Mansoura University, Mansoura, Egypt; e Department of Obstetrics and Gynecology, University of Illinois at Chicago, Chicago, Illinois, USA; f Department of Pathology, University of Illinois at Chicago, Chicago, Illinois, USA; Cornell University

**Keywords:** Leishmania donovani, T cell exhaustion, PD1, PDL-1, autophagy, immunity, immunotherapy, T cell immunity

## Abstract

Leishmania donovani is a causative pathogen of potentially fatal visceral leishmaniasis (VL). Therapeutic agents are available; however, their use is limited because of high cost, serious side effects, and development of antimicrobial resistance. Protective immunity against VL depends on CD4^+^ Th1 cell-mediated immunity. Studies have shown that progression of VL is due to exhaustion of T cells; however, the mechanism involved is not clearly understood. Here, we examined the role of PD1/PDL-1 in the pathogenesis of VL by using a murine model of VL. Our data indicate that L. donovani is able to elicit initial expansion of gamma interferon-producing CD4^+^ Th1 and CD8^+^ T cells at day 7 postinfection (p.i.); however, the frequency of those cells and inflammatory response decreased at day 21 p.i., despite persistence of parasites. Persistent infection-induced expansion of interleukin-10^+^ FOXP3^+^ Treg and CD4^+^ and CD8^+^ T cells expressing PD1. Blocking of PDL-1 signaling *in vivo* resulted in restoration of protective type 1 responses by both CD4^+^ and CD8^+^ T cells, which resulted in a significant decrease in the parasite burden. Mechanistically, PDL-1 blocking inhibited autophagy, a cellular degradation process hijacked by Leishmania to acquire host cell nutrients for their survival. Inhibition of autophagy was marked by decreased lipidation of microtubule-associated protein 1 light chain 3, a marker of autophagosome formation, and P62 accumulation. Together, our findings show for the first time that anti-PDL-1 antibody is an effective therapeutic approach for restoration of effector arms of protective immunity against VL and subsequent parasite clearance.

## INTRODUCTION

Leishmania donovani is one of the causative organisms of visceral leishmaniasis (VL), which is most prevalent on the Indian subcontinent, in East Africa, and in South America. VL is transmitted by the female sand fly and is manifested by chronic fever, hepatosplenomegaly, and pancytopenia and progresses to fatal multiorgan failure if left untreated (1). Control of VL depends on gamma interferon (IFN-γ) production by Th1 CD4^+^ T cells, which promotes protective cell-mediated immunity via several mechanisms, including induction of cytotoxic CD8^+^ T cells that lyse infected cells and activation of macrophage bactericidal functions that clear intracellular parasites (2). In contrast, progression of VL is characterized by the expansion of transforming growth factor beta (TGF-β)- or interleukin-10 (IL-10)-producing T regulatory cells (Tregs) or IL-4-producing Th2 cells (3), which impair intracellular parasite clearance. L. donovani also evades host protective immune mechanisms such as complement-mediated lysis (4) and phagosomal-lysosomal fusion (5). Leishmania lipophosphoglycan also prevents the acidification of phagosomes, which allows promastigotes to differentiate into resistant amastigotes (6). In addition, Leishmania attenuates CD4^+^ T cell priming via negative regulation of TLR2- and TLR4-mediated IL-12 and tumor necrosis factor alpha (TNF-α) production (7, 8), as well as decreasing antigen presentation (9). Current chemotherapies against VL are associated with serious side effects and cannot achieve a sterile cure. Thus, alternative immunotherapy that enhances the different arms of cell-mediated immunity against Leishmania and thus effectively eliminates parasites is warranted.

PD1 and PDL-1 are accessory molecules expressed on T cells and antigen-presenting cells (APCs), respectively (10). Their ligation triggers inhibitory signals that diminish T cell proliferation and cytokine production. Several pathogens exploit the PD1/PDL-1 pathway to suppress innate and adaptive immune responses (11–13). On the other hand, PD1/PDL-1 signals dampen autoimmune responses, and thus it is critical for maintaining effective immune responses against pathogens while avoiding tissue damage caused by dysregulated immune responses and inflammation (14). Blockade of the PD1/PDL-1 pathway during infections with certain pathogens such as Toxoplasma and Plasmodium restored exhausted CD8^+^ T and B cell responses, respectively, controlled parasite reactivation, and prevented death in chronically infected animals (15, 16). However, the effect of blocking PD1/PDL-1 signaling on CD4^+^ T cell responses during L. donovani infection has not been studied.

Autophagy is the mechanism by which cells recycle their cytoplasmic contents in lysosomes. Autophagy plays important roles in the elimination of pathogens, control of inflammation, and adaptive immunity (17). Nevertheless, intracellular pathogens, including Leishmania, have evolved mechanisms to block the autophagic microbicidal defense and subvert the host autophagic machinery for their survival or growth (18). The interaction between autophagy and the PD1/PDL-1 pathway has not been clearly understood.

In this study, we examined the T cell response in VL following murine infection with L. donovani. Our data indicated that L. donovani is able to elicit an initial immune response, followed by deterioration of the inflammatory response and late immunosuppression. Further, *in vivo* blocking of the PD1/PDL-1 pathway with anti-PDL-1 antibodies restored both CD4^+^ and CD8^+^ T cell functions and decreased the parasite burden. Our data also suggest that autophagy inhibition could be a potential mechanism by which anti-PDL-1 antibody therapy exerts its action. These data demonstrate, for the first time, that PD1/PDL-1 blockade in VL is a promising therapeutic approach that is able to control parasite survival and persistence and prevent the development of potentially fatal disease, possibly by blocking autophagy.

## RESULTS

### L. donovani infection is associated with initial T cell activation, which subsides later in the course of infection.

Leishmania is known to exploit the immune mechanisms of the host in order to evade the host immune responses and persist; however, the mechanism by which L. donovani exploits the immune system is not fully understood. In this study, we examined the immune response of mice to L. donovani infection. We selected BALB/c mice for our experiments because they are susceptible to L. donovani infection and develop a VL that recapitulates the disease in humans (19). Wild-type mice were injected with L. donovani promastigotes and body weight and signs of morbidity were monitored. Mice were sacrificed at 7 and 21 days postinfection (dpi), and another group was left for survival studies.

Infection with L. donovani decreased the body weight of infected mice at 7 dpi compared to that of the control group; however, this decrease became significant after 10 weeks postinfection (see Fig. S1A in the supplemental material). L. donovani-infected mice had a slightly greater spleen weight than controls at 7 and 21 dpi (*P* = 0.08) (Fig. S1B). In addition, the total number of splenocytes was significantly lower in infected mice at 21 dpi than the number of infected splenocytes at 7 dpi (*P* = 0.007) (Fig. S1C). The spleens of infected mice showed severely disorganized white pulp and hypertrophy of the red pulp, which was more marked at 21 dpi (Fig. S1D). Staining of liver sections with hematoxylin and eosin (H&E) showed cellular infiltration and formation of granuloma-like structures at both 7 and 21 dpi in infected mice (Fig. S1E); however, there was no clear evidence of apoptotic or necrotic cell death. Quantitative real-time PCR (qRT-PCR) analysis indicated parasite dissemination with a greater burden in the spleens of infected mice at both 7 and 21 dpi than in the bone marrow (BM) (*P* = 0.01) (Fig. S1F). The failure to eliminate parasites at 21 dpi is consistent with signs of morbidity and histological findings and suggests the development of persistent infection.

Protective immunity against VL is mediated by CD4^+^ Th1 cells, where IFN-γ and TNF-α activate microbicidal functions of macrophages to clear the parasites. In addition, generation of Th17 cells is associated with resistance to VL (20). To determine the magnitude and function of antigen-specific immune responses against L. donovani, we analyzed the different T cell subsets in the spleens of infected mice and uninfected controls directly *ex vivo* by flow cytometry. Having comparable controls at both time points justified the use of the data of the control group from the 7-dpi time point. Our data show that L. donovani did not induce significant expansion of total, activated (CD69^+^), or proliferating (Ki67^+^) CD4^+^ T cells in infected mice at 7 dpi compared to the control. Furthermore, failure to clear infection at 21 dpi was associated with a lower number of total and activated CD69^+^ CD4^+^ T cells in infected mice than in control and/or infected mice at 7 dpi (*P* < 0.0001 and *P* = 0.03, respectively) (Fig. S2B to D). Similarly, L. donovani infection did not induce significant expansion of CD8^+^ T cells in infected mice; there was no significant difference in the activation or proliferation of CD8^+^ T cells. Moreover, there were significantly fewer CD69^+^ CD8^+^ T cells in infected mice at 21 dpi than in infected mice at 7 dpi (*P* = 0.006) (Fig. S2E to G). In addition, there was a significantly lower frequency of antigen-specific IFN-γ^+^, TNF-α^+^ CD4^+^ Th1, and IL-17^+^ Th17 cells in infected mice at 21 dpi than in controls and/or infected mice at 7 dpi (*P* = 0.0001, *P* = 0.004, and *P* = 0.003, respectively) (Fig. S2H to J). Similarly, expansion of antigen-specific IFN-γ^+^, but not TNF-α^+^, CD8^+^ Tc1 cells was impaired at 21 dpi compared to that in infected mice at 7 dpi (*P* = 0.003 and *P* = 0.03, respectively) (Fig. S2K and L). Together, these data suggest that persistent L. donovani infection correlates with a decreased frequency of effector antigen-specific CD4^+^ Th1, Th17, and CD8^+^ T cells.

### Induction of regulatory and effector cells expressing inhibitory markers during the course of infection with L. donovani.

**FIG 1:**
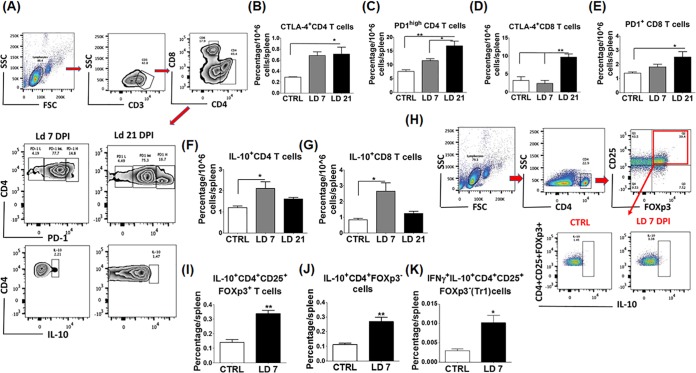
L. donovani upregulates CTLA-4, PD1, and IL-10 expression by different T cell subsets. Splenocytes were harvested from indicated mice at 7 and 21 dpi, fixed, permeabilized, and stained for flow cytometry analysis. (A) Gating strategy for CD4^+^, CD8^+^ T cells, PD1, and IL-10 in CD4^+^ T cells by flow cytometry. SSC, side scatter; FSC, forward scatter. (B) CTLA-4 expression by CD4^+^ T cells is significantly higher in infected mice at 21 dpi than in the control (CTRL). (C) PD1^high^ CD4^+^ T cell levels in infected mice at 7 and 21 dpi are significantly higher than those in control and infected mice at 7 dpi. (D) CTLA-4 expression by CD8^+^ T cells in infected mice at 21 dpi is significantly higher than that in both control and infected mice at 7 dpi. (E) PD1^+^ CD8^+^ T cell levels are significant higher at 21 dpi than in the control. (F) IL-10^+^ CD4^+^ T cell levels at 7 dpi are significantly higher than in the control. (G) IL-10^+^ CD8^+^ T cell levels in infected mice at 7 dpi are significantly higher than those in the control. (H) Gating strategy for Tregs and IL-10 expression by them. (I) The percentage of IL-10^+^ FOXp3^+^ CD4^+^ CD25^+^ cells in infected mice at 7 dpi is significantly higher than in the control. (J) The percentage of IL-10^+^ FOXp3^−^ CD4^+^ cells in infected mice at 7 dpi is significantly higher than in the control. (K) The percentage of IL-10^+^ IFN-γ^+^ CD4^+^ CD25^+^ FOXp3^−^ (Tr1) cells in infected mice at 7 dpi is significantly higher than in the control. *, *P* < 0.05; **, *P* <0.01. Data are presented as the mean ± the SEM of three to five mice per group and are representative of three independent experiments. LD, L. donovani.

The above data suggest that L. donovani may stimulate inhibitory signals that induce either exhaustion or suppression of effector CD4^+^ Th1 cells as an immune evasion mechanism. To test this possibility, we examined the expression of inhibitory markers such as CTLA-4/PD1 on effector T cells as potential mechanisms known to be responsible for T cell exhaustion and/or immunosuppression. Our data demonstrate a significantly higher level of CTLA-4 and PD1 expression on CD4^+^ T cells at 21 dpi than on those of uninfected controls and/or infected mice at 7 dpi (*P* = 0.02 and *P* = 0.003, respectively) (Fig. 1B and C). Similarly, we observed a significantly higher level of CTLA-4 and PD1 expression on CD8^+^ T cells in infected mice at 21 dpi than on those of control and/or infected mice at 7 dpi (*P* = 0.001 and *P* = 0.02, respectively) (Fig. 1D and E).

Induction of Tregs during infection with several intracellular pathogens is associated with suppression of immune responses and ineffective clearance of infection (21). Thus, we examined the frequency of different subsets of Tregs in infected mice at 7 dpi. Our data demonstrated a significant increase in the frequency of IL-10-producing CD4^+^ and CD8^+^ T cells in infected mice at 7 dpi than in those of controls (*P* = 0.02, both); however, the frequency of these cells was similar to that in controls at 21 dpi (Fig. 1F and G). To determine whether IL-10-producing cells are natural Tregs or effector T cells, we examined FOXp3 expression. Our data indicated that L. donovani infection induced expansion of FOXp3^+^ IL-10^+^ CD4^+^ CD25^+^ natural Tregs, as well as FOXp3^−^ IL-10^+^ CD4^+^ effector T cells, in infected mice at 7 dpi compared to control mice (*P* = 0.002 and *P* = 0.007, respectively) (Fig. 1H to J). However, the majority of cells producing IL-10 at 7 dpi were also producing IFN-γ, consistent with type 1 regulatory T (Tr1) cells (*P* = 0.02) (Fig. 1K).

**FIG 2:**
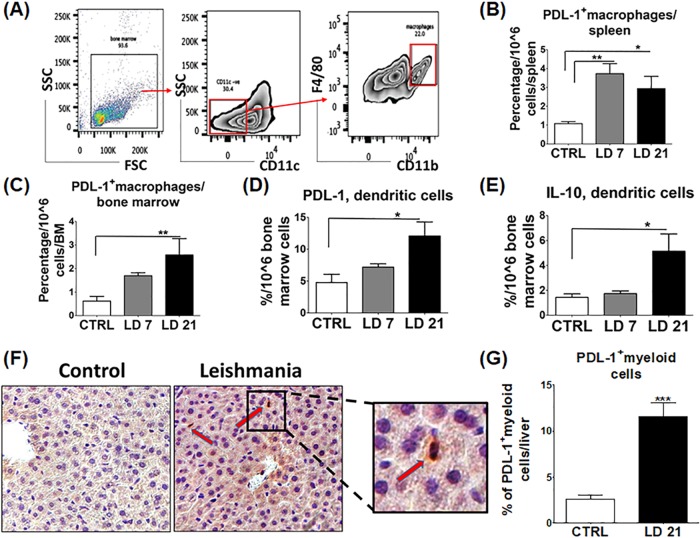
L. donovani infection upregulates PDL-1 in macrophages and DCs. Splenocytes and BM cells were harvested from the mice indicated at 7 and 21 dpi, fixed, permeabilized, and stained for flow cytometry analysis. (A) Gating strategy used for flow cytometry of macrophages (CD11c^−^ CD11b^+^ F4/80^+^) in BM. SSC, side scatter; FSC, forward scatter. (B) The percentage of PDL-1^+^ macrophages in the spleens of infected mice at 7 and 21 dpi is significantly higher than that in the control (CTRL). (C) The percentage of PDL-1^+^ macrophages in BM is significantly higher at 21 dpi than in the control. (D) The percentage of PDL-1^+^ DCs (CD11c^+^ F4/80^−^) in BM is significantly higher at 21 dpi than in the control. (E) The percentage of IL-10^+^ DCs in BM is significantly higher at 21 dpi than in the control. (F) IHC of liver tissue stained with anti-PDL-1 antibody. Red arrows point to positively stained myeloid cells (original magnification, ×63). (G) Quantification of PDL-1-stained myeloid cells at 21 dpi shows a highly significant increase in infected mice compared to those in control mice. *, *P* < 0.05; **, *P* <0.01; ***, *P* <0.001. Data are presented as the mean ± the SEM and are from three independent experiments with three to five mice per group. LD, L. donovani.

Studies have shown that APCs, including dendritic cells (DCs) and macrophages, can suppress T cell responses by providing inhibitory signaling via binding of B7,1/2 or PDL-1 on APCs with the coinhibitor CTLA-4 or PD1 molecules on T cells, respectively (22). To this end, we analyzed PDL-1 expression on DCs (defined as CD11c^+^ F4/80^−^) and macrophages (defined as CD11c^−^ CD11b^+^ F4/80^+^) in the spleen and BM by flow cytometry. The percentage of PDL-1-expressing macrophages in the spleen and BM of infected mice was significantly higher than that in the controls at 7 and/or 21 dpi (*P* = 0.0009 and *P* = 0.008, respectively) (Fig. 2B and C). Similarly, we detected higher percentages of PDL-1-expressing, as well as IL-10-producing, DCs in the BM of infected mice at 21 dpi than in that of controls (*P* = 0.02 and *P* = 0.01, respectively) (Fig. 2D and E). Consistent with flow cytometry data, immunohistochemistry (IHC) staining of liver sections from uninfected and infected mice with an anti-PDL-1 antibody showed positive staining of PDL-1 on myeloid cells in liver sinusoids and blood vessels, including infiltrating circulating macrophages, liver-resident Kupffer cells, and DCs in infected mice (*P* = 0.0005) (Fig. 2F and G).

### Restoration of protective immunity by blocking of PD1/PDL-1 signaling.

**FIG 3:**
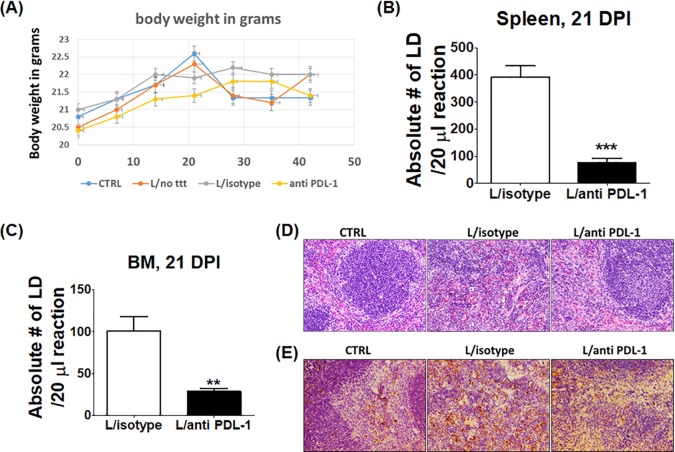
Anti-PDL-1 antibody ameliorates the splenic architecture and decreases the parasite burden dramatically in infected mice. An anti-PDL-1 antibody/isotype control antibody was introduced intraperitoneally into infected mice starting at 7 dpi, and the mice were sacrificed at 21 dpi. (A) The body weight of mice shows no significant change among different groups. (B) qRT-PCR of mouse splenic gDNA showing a highly significant decrease in the parasite burden in anti-PDL-1 antibody-treated mice compared to that in isotype control mice. (C) qRT-PCR analysis of BM gDNA showing a moderately significant decrease in the parasite burden in anti-PDL-1 antibody-treated mice compared to that in isotype control mice. LD, L. donovani. (D) H&E staining of splenic tissues showing the preserved splenic architecture of the anti-PDL-1 antibody-treated group and highly disorganized white pulp in the isotype control spleen (original magnification, ×40). (E) IHC staining of splenic cuts for cleaved caspase 3 showing more staining in the isotype control group than in the control and anti-PDL-1 antibody-treated groups (original magnification, ×40). **, *P* < 0.01; ***, *P* <0.001. Data are presented as the mean ± the SEM of five mice per group and are representative of two independent experiments.

**FIG 4:**
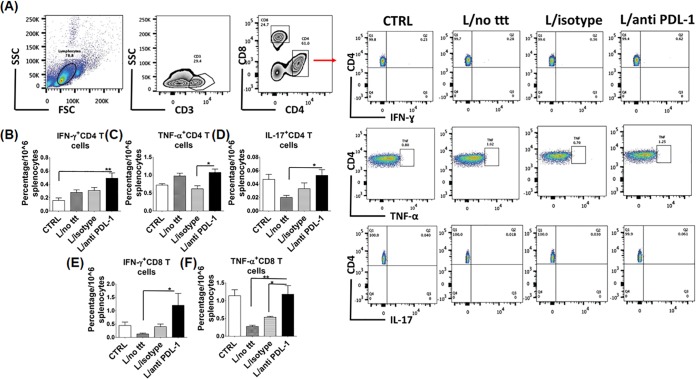
Anti-PDL-1 antibody promotes type 1 response for both CD4^+^ and CD8^+^ T cells. Splenocytes were harvested from the following groups of mice: control (CTRL) noninfected, infected nontreated, infected treated with isotype control antibody, and infected treated with anti-PDL-1 antibody at 21 dpi. Cells were fixed, permeabilized, and stained for flow cytometry analysis. (A) Gating strategy for flow cytometry of CD4^+^ and CD8^+^ T cells and IFN-γ, TNF-α, and IL-17 in CD4^+^ T cells. SSC, side scatter; FSC, forward scatter. (B) The percentage of IFN-γ^+^ CD4^+^ T cells is significantly higher in the anti-PDL-1 antibody-treated group than in the control group. (C) The percentage of TNF-α^+^ CD4^+^ T cells is significantly higher in the anti-PDL-1 antibody-treated group than in the isotype control groups. (D) The percentage of IL-17^+^ CD4^+^ T cells is significantly higher in the anti-PDL-1 antibody-treated group than in the infected nontreated group. (E) The percentage of IFN-γ^+^ CD8^+^ T cells is significantly higher in the anti-PDL-1 antibody-treated group than in the infected nontreated group. (F) The percentage of TNF-α^+^ CD8^+^ T cells is significantly higher in the anti-PDL-1 antibody-treated group than in the infected nontreated and isotype control groups. *, *P* < 0.05; **, *P* <0.01. Data are presented as the mean ± the SEM and are from three independent experiments with three to five mice per group.

We hypothesized that persistent L. donovani infection is due to defective expansion of effector CD4^+^ Th1 cells and IFN-γ-producing CD8^+^ T cells during the late stage of infection secondary to PD1/PDL-1-mediated immunosuppression or exhaustion. To test this hypothesis, we blocked PDL-1 on APCs in infected mice as described in Materials and Methods. Infected mice were classified into three groups, i.e., those infected and treated with anti-PDL-1 antibody (L/anti-PDL-1) and two control groups consisting of those infected and not treated (L/no ttt) and those infected and treated with an isotype control antibody (L/isotype). Blocking of PD1/PDL-1 signaling did not affect body weight during the course of infection (Fig. 3A); however, treatment with anti-PDL-1 antibody resulted in effective clearance of parasites from the spleen and BM compared to the isotype control (*P* < 0.0001 and *P* = 0.003, respectively) (Fig. 3B and C). Further, blocking of PDL-1 restored the splenic architecture, as marked by the presence of well-organized white pulp and absence of amastigotes (Fig. 3D). In addition, the spleens of anti-PDL-1 antibody-treated mice had fewer caspase 3-positive stained cells than did those of controls, which suggests that blocking of PD1/PDL-1 signaling decreased the apoptosis of leukocytes (Fig. 3E). Further, blocking of PDL-1 signaling resulted in a significantly greater percentage of CD4^+^ Th1 cells producing IFN-γ or TNF-α, Th17 cells producing IL-17, and CD8^+^ Tc1 cells producing IFN-γ or TNF-α in the anti-PDL-1 antibody-treated group than in the control groups (*P* = 0.01, *P* = 0.007, *P* = 0.03, *P* = 0.02, and *P* = 0.008, respectively) (Fig. 4A to F).

### Anti-PDL-1 antibody enhanced T cell priming functions of macrophages.

**FIG 5:**
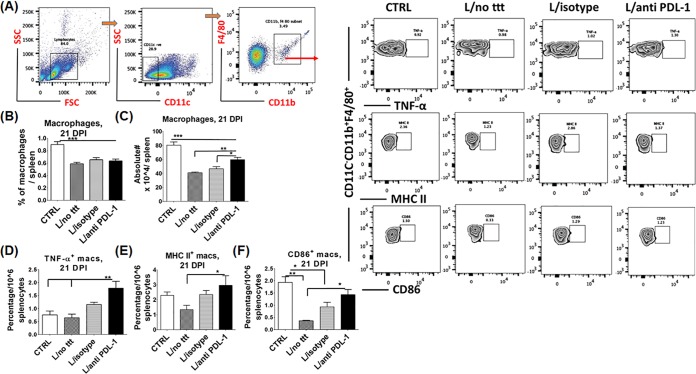
Anti-PDL-1 antibody enhances T cell priming by macrophages. Splenocytes were harvested from the following groups of mice: control (CTRL) noninfected, infected nontreated, infected treated with isotype control antibody, and infected treated with anti-PDL-1 antibody at 21 dpi. Cells were fixed, permeabilized, and stained for flow cytometry analysis. (A) Gating strategy for macrophages (CD11c^−^ CD11b^+^ F4/80^+^) and their expression of TNF-α, CD86, and MHC class II in the spleen by flow cytometry. SSC, side scatter; FSC, forward scatter. (B) Decreased percentage of macrophages in the spleens of infected groups compared to that in negative controls. (C) The absolute number of macrophages in the anti-PDL-1 antibody-treated group is significantly higher than that the infected nontreated and isotype control groups. (D) The percentage of TNF-α^+^ macrophages is significantly higher in the anti-PDL-1 antibody-treated group than in the negative- and positive-control groups. (E) The percentage of MHC II^+^ macrophages in the spleen is significantly higher in the anti-PDL-1 antibody-treated group than in the infected nontreated group. (F) The percentage of CD86^+^ macrophages in the anti-PDL-1 antibody-treated group is significantly greater than that in the infected nontreated group. *, *P* < 0.05; **, *P* <0.01; ***, *P* <0.001. Data are from three independent experiments and are presented as the mean ± the SEM of three to five mice per group.

Restoration of T cell functions in anti-PDL-1 antibody-treated mice could be due to enhanced T cell priming functions of APCs, mainly macrophages as the main target cells for Leishmania, and/or decreased inhibitory or immunosuppressive signals. To examine these possibilities, we first measured the effect of treatment on macrophage survival, activation, and effector functions (e.g., upregulation of major histocompatibility complex (MHC) class II and costimulatory molecules and production of TNF-α). We found that infected mice treated with anti-PDL-1 antibodies had a significantly higher number of macrophages, mainly those producing TNF-α, than controls (*P* < 0.0001 and *P* = 0.001, respectively) (Fig. 5A to D). In terms of antigen presentation capacity, blocking of PDL-1 increased the percentage of activated macrophages expressing high levels of MHC class II and costimulatory molecule CD86 (i.e., signals I and II for T cell priming) compared to that in infected nontreated mice (*P* = 0.03 and *P* = 0.002, respectively) (Fig. 5E and F).

Next, we examined the effect of PDL-1 blocking on T cell responses at 21 dpi. The percentage of immunosuppressive IL-10 or CTLA-4 in CD4^+^ or CD8^+^ T cells was not significantly different in the anti-PDL-1 antibody-treated and control groups at 21 dpi (data not shown).

### Anti-PDL-1 antibody-induced memory T cell responses.

To determine whether blocking of PDL-1 signaling enhances the memory responses against Leishmania and provides long-term protective immune responses, we first analyzed the frequency of effector memory (EM) CD44^+^ CD62L^−^ and central memory (CM) CD44^+^ CD62L^+^ CD4^+^ and CD8^+^ T cells at 21 dpi. Our data showed that anti-PDL-1 antibody treatment enhanced the expansion of CD44^+^ CD62L^−^ EM CD4^+^ T cells compared to the infected nontreated group (*P* = 0.01) (Fig. S3B). Additionally, it enhanced the expansion of CD44^+^ CD62L^−^ EM CD8^+^ T cells compared to that in the negative- and positive-control groups (*P* = 0.001) (Fig. S3C). The number of CD44^+^ CD62L^+^ CM CD4^+^ and CD8^+^ T cells at 21 dpi was higher in infected untreated mice than in infected mice treated with anti-PDL-1 or isotype control antibody; however, the number of CM CD8^+^ T cells in the anti-PDL-1 antibody-treated group was higher than that of the isotype control group (*P* = 0.0002) (Fig. S3D and E).

Next, we investigated the effect of anti-PDL-1 antibody on the parasite burden at 42 dpi. Blocking of PDL-1 signaling significantly decreased the parasite burden in the spleen and the BM compared to that in the isotype control group, as demonstrated by qRT-PCR analysis (*P* < 0.0001 and *P* = 0.001, respectively) (Fig. S4A and B). H&E staining also revealed an organized architecture of the white pulp of the spleen in anti-PDL-1 antibody-treated mice (Fig. S4C), while isotype control mice showed disorganized lymphoid follicles consistent with hyperactivation. Finally, IHC staining suggested decreased apoptosis in the anti-PDL-1 antibody-treated group, as marked by less cleaved caspase 3 staining than in the isotype control group (Fig. S4D).

### Autophagy inhibition is a mechanism of action used by anti-PDL-1 antibody.

**FIG 6:**
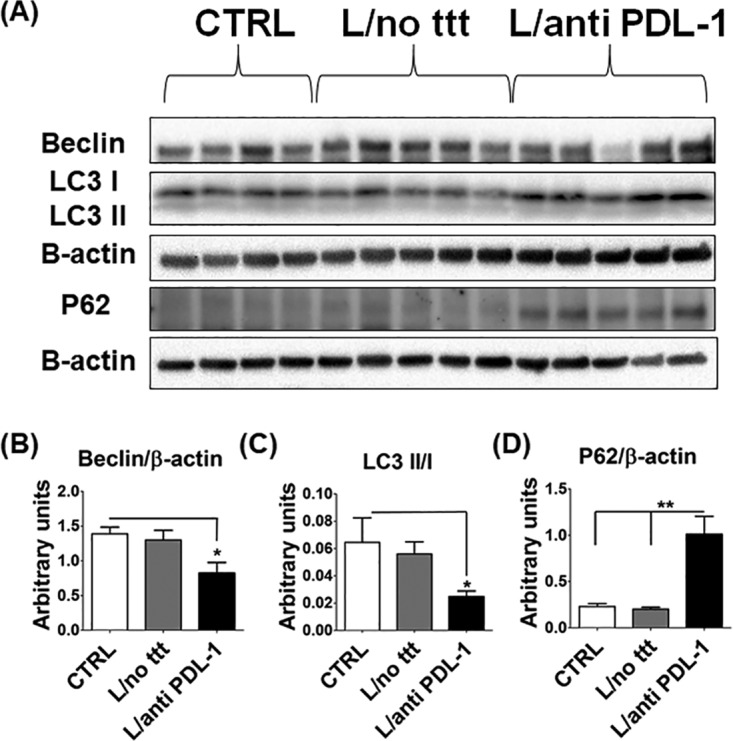
Autophagy inhibition is a possible mechanism of action of the anti-PDL-1 antibody. BM cells from uninfected and infected mice not treated or treated with anti-PDL-1 antibody were harvested, and autophagy markers were analyzed by immunoblotting. (A) Expression of LC3I, beclin, and P62. β-Actin was used as an internal control (CTRL). (B to D) Densitometry data showing that the anti-PDL-1 antibody-treated group has less beclin (B) and a lower LC3II-to-LC3I ratio (C) but more P62 (D) than the control groups. *, *P* < 0.05; **, *P* <0.01. Data are presented as the mean ± the SEM and are from three independent experiments with four or five mice per group.

Autophagy acts as an innate immune defense mechanism against intracellular pathogens. On the other hand, studies using cutaneous and VL models showed that Leishmania exploits autophagy to obtain nutrients to survive and multiply within membrane-bound compartments (23, 24). These studies suggested that autophagy is a pathogen-friendly mechanism during Leishmania infection. Since anti-PDL-1 antibody treatment resulted in effective elimination of L. donovani and enhanced T cell responses, we hypothesized that anti-PDL-1 antibody might have suppressed autophagy. To measure autophagy induction, we assessed the expression of beclin-1 (the initial autophagy protein that binds to other autophagy proteins, Atgs, to form the phagophore), as well as the ratio of LC3II to LC3I (LC3I is a cytosolic nonlipidated molecule that is recruited to the phagophore and undergoes proteolytic cleavage and lipidation, yielding LC3II, which forms the autophagosome). To this end, BM tissues were harvested at 21 dpi from uninfected and infected mice that were either left untreated or treated with anti-PDL-1 antibody, and the expression level of beclin-1 and LC3II-to-LC3I ratio in BM lysates were assessed by immunoblotting with anti-beclin and anti-LC3 antibodies, respectively. We chose BM because it is one of the major sites of L. donovani infection in humans and mice and is highly enriched with progenitors of mononuclear phagocytic cells, the major target cells for Leishmania. We found that treatment of infected mice with anti-PDL-1 antibody inhibited autophagy induction in BM cells, as marked by a lower beclin-1 level and LC3II-to-LC3I ratio than in uninfected controls (*P* = 0.02 and *P* = 0.05, respectively) (Fig. 6A to C). We also analyzed autophagic flux in BM tissue lysates by analyzing the level of p62/SQSTM1, a selective autophagy adaptor/receptor that binds to ubiquitinated proteins and damaged organelles to target them to autophagosomal-lysosomal compartments for degradation. The total cellular p62 expression level inversely correlates with the autophagic flux and activity (25). Compared to the control groups, inhibition of autophagosome formation in anti-PDL-1 antibody-treated mice was associated with accumulation of P62 (*P* = 0.0007) (Fig. 6D), suggesting a block of autophagy flux.

## DISCUSSION

The outcome of infection with L. donovani and the ensuing immune responses vary in different studies because of the differences in the host genetic background, parasite strains, route of infection, and inoculum dose (26). Previous studies indicated that immune dysfunction due to CD8^+^ T cell exhaustion or suppression is responsible for chronic infection and susceptibility to VL (27). However, the role of CD4^+^ T cells in VL, and whether these cells undergo exhaustion during L. donovani infection remains elusive. In this study, we investigated the magnitude and function of both CD4^+^ and CD8^+^ T cells, which are major components of protective cell-mediated immunity to Leishmania infection. We demonstrated that L. donovani is able to induce an initial suboptimal CD4^+^ and CD8^+^ T cell immune responses at 7 dpi in the spleen; however, this response becomes weak and ineffective in the elimination of parasites at 21 dpi (Fig. S2). Furthermore, we showed that this dysfunctional adaptive immunity was associated with heightened expression of inhibitory molecules, including CTLA-4 and PD1, as well as expansion of Tregs (Fig. 1). More importantly, blocking of PDL-1 *in vivo* decreased the parasite burden (Fig. 3B and C) and enhanced the magnitude of protective type 1 CD4^+^ and CD8^+^ T cell-mediated immune responses (Fig. 4), as well as the T cell activation function of macrophages (Fig. 5C to F). We attributed the action of anti-PDL-1 antibody, in part, to autophagy inhibition (Fig. 6). Thus, our data introduce anti-PDL-1 antibody as an important effective and protective immunotherapy against VL.

IL-10 has a major role in the regulation of inflammation, disease development, and pathogen persistence (28–30), as it produces its effect by disabling Th1 cell-type responses, preventing macrophage activation, and decreasing their production of key Th1 cytokines such as IL-12 and TNF-α (28, 31). Thus, our data showing expansion of IL-10-producing effector CD4^+^ and CD8^+^ cells and Tregs, as well as by DCs, during the course of L. donovani infection could account for defective parasite killing by macrophages at later stages of infection (Fig. 1F to K and 2E). We also found that increased IL-10 production is associated with decreased expansion of TNF-α-producing CD4^+^ and CD8^+^ T cells at 21 dpi (Fig. S2I and L). Since TNF-α has important roles in macrophage activation and intracellular parasite clearance (32), it is possible that IL-10 promotes parasite persistence via inhibition of TNF-α secretion by T and non-T cells. These data are consistent with other studies showing that depletion of IL-10 enhances TNF-α production, reduced pathology, and enhanced resistance to L. donovani infection (33, 34) and that IL-10 receptor blockade is a potential immunotherapeutic approach in L. donovani infection (31).

The PD1/PDL-1 pathway is a key signaling pathway that ensures a critical balance between the stimulatory and inhibitory signals needed for effective immune responses to microbes and for self-tolerance (14). The PD1/PDL-1 pathway is exploited by several microorganisms that cause chronic infections to weaken the immune response and support infection (35). Consistent with other studies that used Leishmania infantum and L. donovani models (2, 10), we found that PD1 and PDL-1 were upregulated on T cells and macrophages, respectively (Fig. 1C and E and 2B to D, F, and G). Importantly, we demonstrate here that treatment of infected mice with anti-PDL-1 antibodies that block the interaction of PDL-1 on APCs with a PD1 receptor on T cells ameliorated the splenic architecture, decreased apoptosis in white and red pulp, and decreased the parasite burden dramatically in both the spleen and BM (Fig. 3B to E). Blocking of PD1/PDL-1 signaling also enhanced the production of proinflammatory cytokines, including IFN-γ, TNF-α, and IL-17, by CD4^+^ and CD8^+^ T cells (Fig. 4B to F). The improvement of the cytokine profile of protective antigen-specific CD4^+^ and CD8^+^ T cells producing IFN-γ was surprising, since two similar studies showed improved survival of antigen-specific CD8^+^ T cells but not the cytokine profile after PD1/PDL-1 blockade in L. infantum and L. donovani models (2, 10). Thus, our data suggest that restoration of antigen-specific T cell responses during VL can be achieved by targeting the PDL-1 pathway at early, but not at later, stages of infection, as suggested by other studies. Notably, blocking of PD1/PDL-1 at both early and late stages of infection (21 and 42 dpi) was associated with induction and maintenance of EM CD4^+^ and CD8^+^ T cell responses (Fig. S3 and S4 and data not shown). Together, our data suggest that blocking of PDL-1 signaling improved not only primary immune responses against L. donovani but also memory responses. Further studies will examine the mechanism by which anti-PDL-1 antibody immunotherapy influences the recall response against L. donovani and other agents causing VL.

How blocking of PD1/PDL-1 signaling restores protective primary CD4^+^ and CD8^+^ T cell responses is not clearly understood. Our data suggest that blocking of PDL-1 signaling more likely inhibits the apoptosis of immune and host cells, as marked by lower expression of cleaved/active caspase 3 in treated/infected mice than in controls (Fig. 3E). This conclusion is consistent with recent studies showing reduced apoptosis of T cells in mice infected with other intracellular protozoa such as *Toxopla*sma and treated with anti-PDL-1 antibodies (15). The improvement in T cell functions was reflected in macrophages, which exhibited increased absolute numbers, as well as increased markers related to activation and antigen presentation (Fig. 5C to F). Other studies of malignant glioma and melanoma have shown that this treatment restores CD4^+^ and CD8^+^ effector T cells by abrogation of the immunosuppressive effect of Tregs which express these inhibitory markers and produce immunosuppressive IL-10 cytokine (36, 37).

Although anti-PDL-1 antibodies confer a protective response against VL, as shown in this study, one has to consider the role of PD1/PDL-1 in the prevention of autoimmunity and immunopathology. In that regard, our data indicate that early treatment with anti-PDL-1 antibody was not only able to induce protective immunity but also was able to preserve tissue integrity, as marked by the presence of organized architecture of the white pulp in the spleen of anti-PDL-1 antibody-treated mice compared to untreated infected controls (Fig. S4C). The mechanism by which anti-PDL-1 antibody treatment prevents immunopathology and tissue damage in our model remains elusive. However, it is possible that decreased expansion of TNF-α^+^ CD8^+^ T cells in the treated group compared to that in controls may account for decreased host cell death and tissue damage (38).

Several Leishmania species, such as L. major, L. infantum, L. amazonensis, and L. donovani (23, 24, 39, 40), induce autophagy as a mechanism of immune evasion, as it attenuates T cell responses (23). Recent studies have shown that Leishmania species exploit the autophagy process and induce autophagosome formation to obtain nutrients for their intracellular survival and/or replication while inhibiting lysosomal acidification and degradation by lysosomal enzymes (41, 42). Our data (Fig. 6) are consistent with these studies, as we show, for the first time, that treatment of infected mice with anti-PDL-1 antibody inhibited autophagosome formation in BM tissues, suggesting that the antiparasite effect of anti-PDL-1 antibody treatment could be due to attenuated autophagy induction. The accumulation of p62 in anti-PDL-1 antibody-treated mice could also be due to block of autophagy flux since cellular p62 expression levels inversely correlate with the autophagic flux and lysosomal activity. As BM tissues contain a mixture of pluripotent stem cells and myeloid and lymphoid progenitors, it is not yet clear in which cell subsets the inhibition of autophagy induction and flux occurs. However, the correlation between autophagy inhibition in BM tissues and the decreased parasite burden in BM suggests that autophagy inhibition in anti-PDL-1-treated mice is more likely to occur in the progenitors of myeloid cells or mature mononuclear phagocytic cells as major target cells for Leishmania. Nevertheless, further studies are required to dissect autophagy flux by using lysosomal acidification inhibitors. Together, our data suggest that the decreased parasite burden in anti-PDL-1 antibody-treated mice could be due to inhibition of autophagosome formation as a source of nutrients for the parasite's survival.

In conclusion, our study provides strong evidence that blocking of the PD1/PDL-1 pathway is a promising effective immunotherapeutic strategy against VL that restores protective antigen-specific CD4^+^ and CD8^+^ type 1 responses without inducing autoimmunity or immunopathology. Mechanistically, this study suggests that autophagy inhibition could be a potential mechanism by which anti-PDL-1 antibody promotes protective immunity against VL.

## MATERIALS AND METHODS

### Ethics statement.

The animal experiments in this study were approved by the Institutional Animal Care and Use Committee of Augusta University, Augusta, GA.

### Mice, parasites, and experimental infections.

Six- to 8-week-old inbred female BALB/c mice were purchased from the Jackson Laboratory (Bar Harbor, ME, USA) and used for all experiments. All animals were housed under specific-pathogen-free conditions at the Animal Research Facility, Augusta University. L. donovani promastigotes (30030; ATCC, Manassas, VA, USA) were grown in M199 medium (12-109F; Lonza, Walkersville, MD, USA) supplemented with 10% fetal bovine serum (10082-147; Gibco, Waltham, MA, USA) and 0.2% hemin solution, the pH was adjusted to 7, and the parasites were grown in a standard incubator at 26°C. Stationary-phase promastigotes were centrifuged at 1,000 × *g* (Sorvall ST 16 centrifuge; Thermo Scientific, Waltham, MA, USA), washed twice with phosphate-buffered saline (PBS), counted with a hemocytometer, and then resuspended in PBS at a concentration of 5 × 10^7^/ml. Mice were infected via the intravenous route with 10^7^ promastigotes/mouse and monitored daily for signs of illness and survival.

### *In vivo* administration of antibodies.

Anti-mouse PDL-1 antibody (CD274) and rat IgG2b isotype control antibodies (124328 and 400664; BioLegend, San Diego, CA, USA) were administered intraperitoneally at 400 μg/mouse in four divided doses at 7, 10, 14, and 17 dpi.

### Flow cytometry.

Spleens and BM were harvested, and single-cell suspensions were made with a 40- to 70-μm cell strainer. Cells were used as direct *ex vivo*, counted, fixed, and permeabilized with the BD Cytofix/Cytoperm kit (554714; BD Biosciences, San Jose, CA, USA). FcRs were blocked with a monoclonal antibody (14-9161-71; eBioscience, Cleveland, OH, USA) against mouse cell surface antigens CD16 and CD32 for 15 min and then stained according to standard protocols. The following antibodies (with the specific catalog number in parentheses) were purchased from BioLegend (San Diego, CA, USA) and used at the concentrations recommended by the manufacturer: anti-CD3 (100221, 100218), anti-CD4 (100406), anti-CD8a (100708), anti-CD69 (104510), anti-CD25 (101904), anti-CD44 (103005), anti-CD62L (104427), anti-CD11c (117318), anti-CD11b (101212), anti-F4/80 (123118), anti-Ki69 (652425), anti-PD1 (135210), anti-IL-10 (505009, 505008, 505036), anti-IFN-γ (505825, 505822), anti-TNF-α (506322), anti-CD152 (anti-CTLA-4) (106313), anti-IL-17 (506943), anti-PDL-1 (124308), and anti-CD86 (105006). Anti-FOXp3 (17-5773-82) and anti-MHC class II (12-5322) antibodies were purchased from eBioscience (Cleveland, OH, USA). Lymphocytes and macrophages were gated on the basis of forward and side scatter parameters, and 100,000 events were collected with a flow cytometer (BD Systems, San Jose, CA, USA). Data were analyzed with FlowJo 0.7 (FlowJo Software LLC, Ashland, OR, USA).

### Protein extraction and Western blotting.

Cells extracted from BM were resuspended in radioimmunoprecipitation assay buffer with protease inhibitors (P8340; Sigma, St. Louis, MO, USA), processed with a Sonic Dismembrator (FB-120; Fisher Scientific, Waltham, MA, USA), and centrifuged at 12,000 rpm for 10 min at 4°C, and the supernatant was collected. Protein concentration was measured with protein assay reagent (500-0006; Bio-Rad, Hercules, CA, USA), and the optic density was measured with Synergy HT plate reader (BioTek, Winooski, VT, USA). The proteins were then separated by 4 to 20% SDS-PAGE (456-1096; Bio-Rad, Hercules, CA, USA) and transferred onto a polyvinylidene difluoride membrane (IPVH00010; Millipore, Temecula, CA, USA). Blocking treatment of the membrane was done with nonfat dry milk (170-6404; Bio-Rad, Hercules, CA, USA), and the membrane was incubated at 4°C overnight with the following antibodies diluted in accordance with the manufacturer's instructions: anti-LC3A/B rabbit monoclonal antibody, anti-beclin-1 rabbit monoclonal antibody (12741 and 3495, respectively; Cell Signaling, Danvers, MA, USA), anti-SQSTM1/P62 mouse monoclonal antibody (ab56416; Abcam, Cambridge, MA, USA), and anti-β-actin mouse monoclonal antibody (A2228; Sigma, St. Louis, MO, USA). Subsequent to washing, the horseradish peroxidase-linked anti-rabbit and anti-mouse IgG 7074 and 7076 secondary antibodies (Cell Signaling, Danvers, MA, USA), respectively, were added and the membrane was incubated at room temperature for 1 h. Finally, the membrane was developed by using an enhanced chemiluminescence reagent (34076; Thermo Scientific, Waltham, MA, USA). The developed film was scanned with the ChemiDoc imaging system (Bio-Rad, Hercules, CA, USA) and densitometry was performed with Image Lab software (Bio-Rad, Hercules, CA, USA).

### Histopathology.

Livers and spleens were fixed in 10% paraformaldehyde, paraffin wax embedded, sectioned, and stained with H&E, as well as IHC with anti-cleaved caspase 3 antibody (9661; Cell Signaling, Danvers, MA, USA) and anti-PDL-1 antibody (4059; ProSci, Poway, CA, USA); this was followed by detection with a biotin-labeled anti-rabbit antibody and staining with Vectastain ABC kit purchased from Vector Laboratories (Burlingame, CA, USA). Stained splenic imprints were obtained with Giemsa stain (GS500; Sigma, St. Louis, MO, USA). Slides were examined by EVOS (Thermo Fisher Scientific, Waltham, MA, USA) and Zeiss Axioplan microscopy (Carl Zeiss Microscopy LLC, Thornwood, NY, USA).

### qRT-PCR.

DNA was extracted from the spleens and BM of mice with the DNeasy blood and tissue kit (69506; Qiagen, Germantown, MD, USA) in accordance with the manufacturer's instructions. Real-time PCR was performed with iTaq Universal SYBR green supermix from Bio-Rad (Raleigh, NC, USA). We used the following primers, which target kinetoplastid DNA: *JW11* (forward), 5′-CCTATTTTACACCAACCCCCAGT-3′; *JW12* (reverse), 5′-GGGTAGGGGCGTTCTGCGAAA-3′ (43). The following protocol was used: an initial denaturation cycle of 94°C for 5 min, followed by 40 amplification cycles of 94°C for 15 s and 58°C for 30 s on a CFX Connect real-time system (Bio-Rad, Hercules, CA, USA). A standard curve was created by using genomic DNA (gDNA) extracted from L. donovani promastigotes grown in culture and was used to calculate the absolute number of L. donovani DNA copies per PCR.

### Statistical analysis.

Statistical analysis was done with GraphPad Prism 5 (GraphPad Software Inc., San Diego, CA, USA). Comparisons of two groups were made with the Student *t* test. For comparison of multiple experimental groups, we used one-way analysis of variance with Bonferroni's *post hoc* test. Data are presented as the mean ± the standard error of the mean (SEM). *P* < 0.05 was taken as evidence of statistical significance.

## Supplementary Material

Supplemental material
