# Supplementary material for: PDL-1 Blockade Prevents T Cell Exhaustion, Inhibits Autophagy, and Promotes Clearance of Leishmania donovani
Source: Infect Immun. 2018 May 22;86(6):e00019-18. doi: 10.1128/IAI.00019-18 (PMC5964517; doi:10.1128/IAI.00019-18)
Supplement: Supplemental material [file supp_86_6_e00019-18__index.html]

Supplemental material 

# PDL-1 Blockade Prevents T Cell Exhaustion, Inhibits Autophagy, and Promotes Clearance of Leishmania donovani

## Supplemental material

- Supplemental file 1 -

  Fig. S1. Susceptibility of BALB/c mice to *L. donovani* infection. Fig. S2. *L. donovani* infection causes defective expansion of T cells of Th1 and Th17 phenotypes at late stage of infection. Fig. S3. Anti-PDL-1 promotes effector memory CD4+ and CD8+ T cells. Fig. S4. Anti-PDL-1 continues its antileishmanial effect at 42 dpi without causing tissue damage.

  PDF, 1.2M
