# Supplementary material for: PDL-1 Blockade Prevents T Cell Exhaustion, Inhibits Autophagy, and Promotes Clearance of Leishmania donovani
Source: Infect Immun. 2018 May 22;86(6):e00019-18. doi: 10.1128/IAI.00019-18 (PMC5964517; doi:10.1128/IAI.00019-18)
Supplement: Supplemental material [file IAI.00019-18_zii999092424s1.pdf]

Figure S1

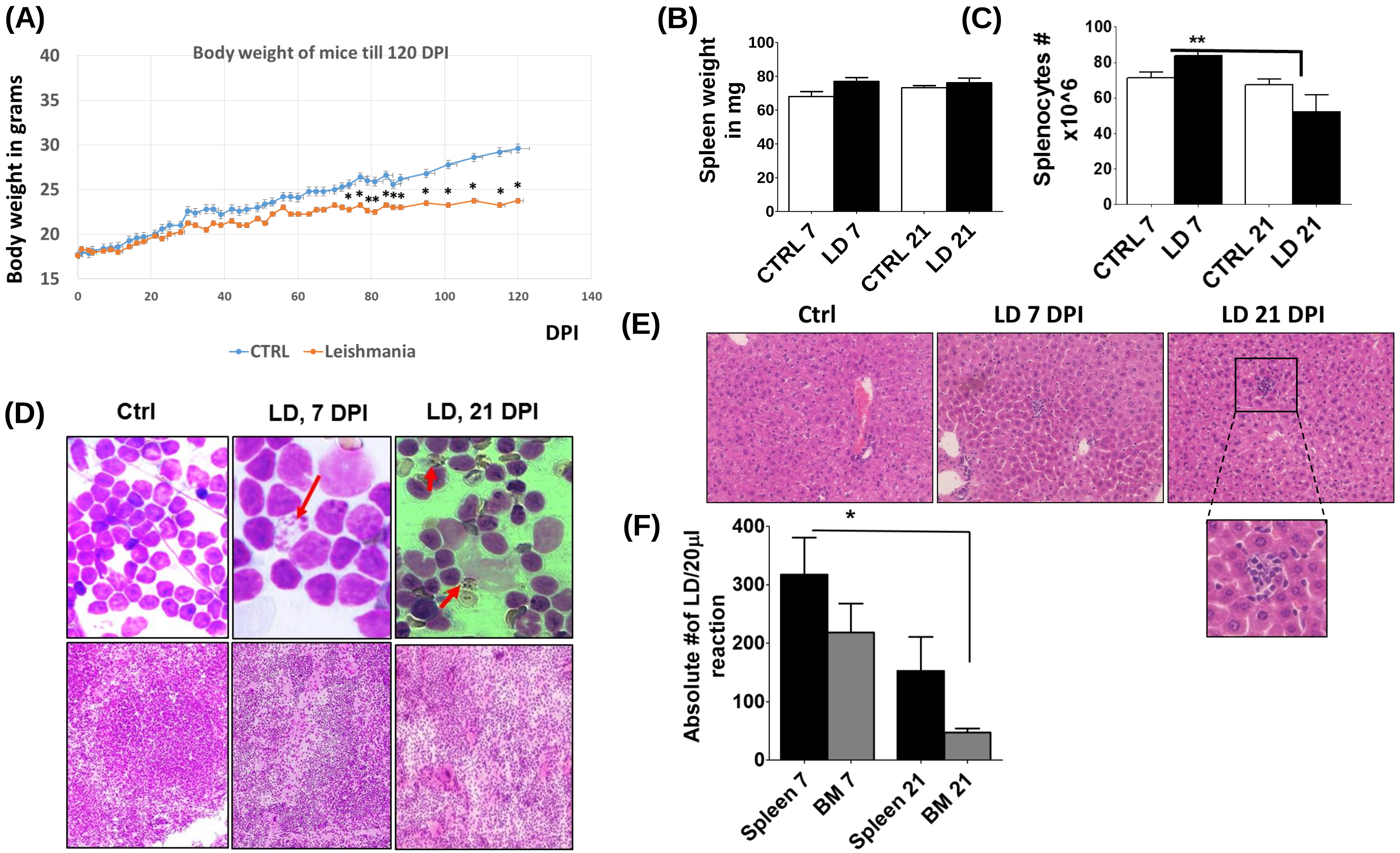
(A)
Body weight of mice till 120 DPI
Body weight in grams
40
35
30
25
20
15
0
20
40
60
80
100
120
140
DPI
CTRL
Leishmania
(B)
Spleen weight in mg
100
80
60
40
20
0
CTRL 7
LD 7
CTRL 21
LD 21
(C)
Splenocytes # x10^6
100
80
60
40
20
0
**
CTRL 7
LD 7
CTRL 21
LD 21
(D)
Ctrl
LD, 7 DPI
LD, 21 DPI
(E)
Ctrl
LD 7 DPI
LD 21 DPI
(F)
Absolute #of LD/20µl reaction
400
300
200
100
0
*
Spleen 7
BM 7
Spleen 21
BM 21

Fig S1. Susceptibility of BALB/c mice to *L. donovani* infection. BALB/c mice were infected i.v., and signs of morbidity were examined. **(A)** Body weight of infected mice shows significant decrease at 10 weeks p.i. **(B)** Splenic weight in infected mice shows non-significant increase than the controls. **(C)** The number of splenocytes in infected mice at 21 DPI showing significant decrease compared to infected mice at 7 DPI. **(D)** Giemsa (upper panel) and H&E staining (lower panel) of splenic imprints and tissue sections from spleen of all mice groups at 7 and 21 DPI. Data demonstrate *Leishmania* amastigotes (Red arrow-upper panel) (original magnification 63×) and highly disorganized splenic white pulp with marked decrease in the cell density in the red pulp at 21 DPI (lower panel) (original magnification 40×). Insets show intracellular amastigotes in infected mice spleens. **(E)** H&E staining of liver section showing *Leishmania* granuloma (original magnification 40×). **(F)** qRT-PCR for gDNA extracted from spleen and bone marrow of infected mice showing higher parasitic burden in the spleen than the bone marrow **.** * and ** indicate $p<0.05$, <0.01 respectively. Data are presented as mean ± SEM and are from three independent experiments with 3-5 mice/group.

Figure S2

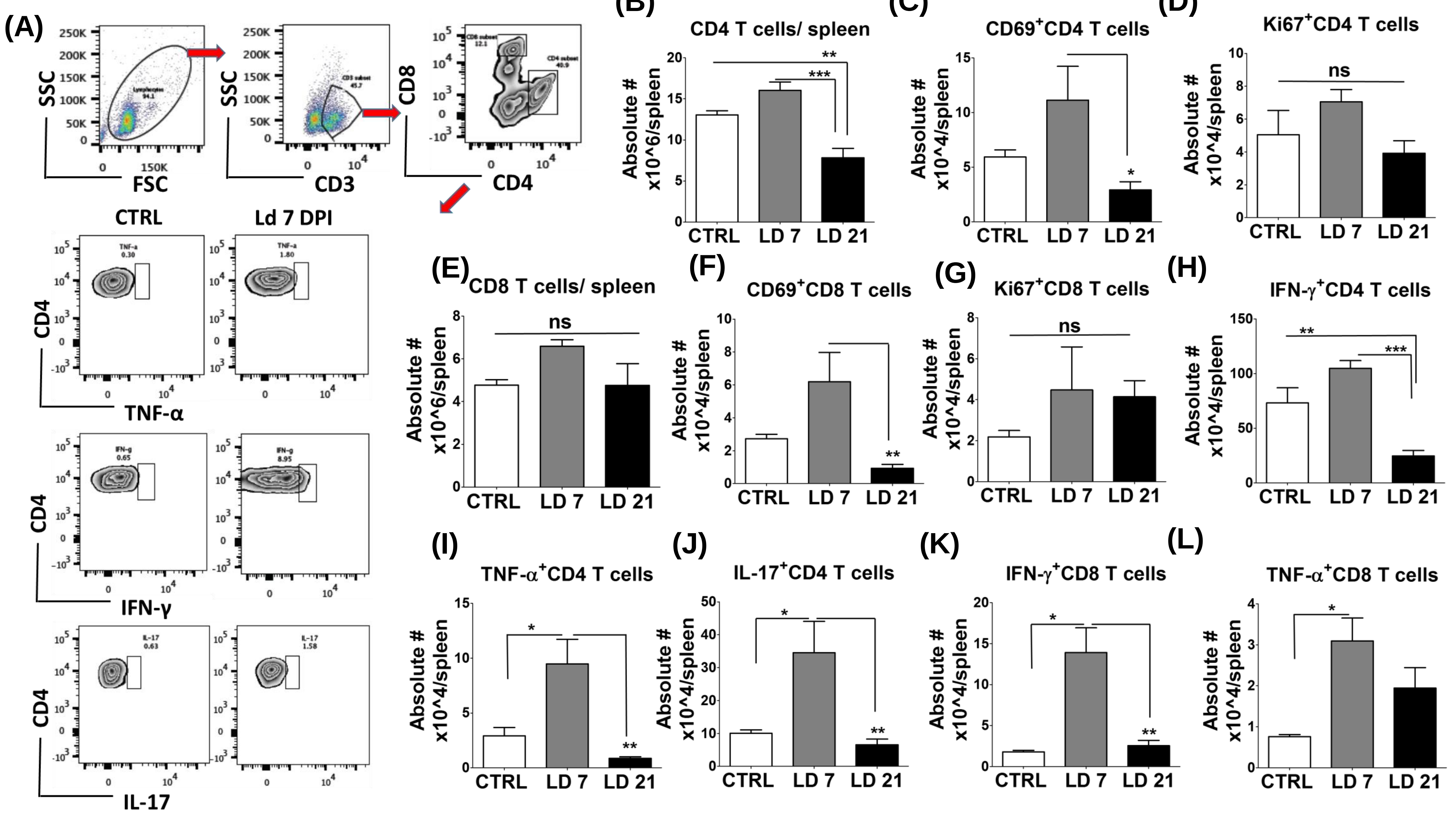
(A)
SSC
FSC
Lymphocytes 94.1
SSC
CD3
CD3 subset 45.7
CD8
CD4
CD8 subset 12.1
CD4 subset 40.9
CTRL
Ld 7 DPI
CD4
TNF-α
TNF-a 0.30
TNF-a 1.80
CD4
IFN-γ
IFN-g 0.65
IFN-g 8.95
CD4
IL-17
IL-17 0.63
IL-17 1.58
(B)
CD4 T cells/ spleen
Absolute # x10^6/spleen
CTRL
LD 7
LD 21
(C)
CD69+CD4 T cells
Absolute # x10^4/spleen
(D)
Ki67+CD4 T cells
ns
(E)
CD8 T cells/ spleen
Absolute # x10^6/spleen
ns
(F)
CD69+CD8 T cells
(G)
Ki67+CD8 T cells
ns
(H)
IFN-γ+CD4 T cells
(I)
TNF-α+CD4 T cells
(J)
IL-17+CD4 T cells
(K)
IFN-γ+CD8 T cells
(L)
TNF-α+CD8 T cells

Fig S2. *L. donovani* infection causes defective expansion of T cells of Th1 and Th17 phenotypes at late stage of infection. Splenocytes were harvested from the indicated mice at 7 and 21 DPI, fixed, permeabilized and stained for flow cytometry analysis. **(A)** Gating strategy for $CD4^+$, $CD8^+$ T cells, TNF-α, IFN-γ and IL-17 in $CD4^+$ T cells by flow cytometry. **(B)** The absolute number of $CD4^+$ T cells of infected mice at 21 DPI shows significant decrease compared to control and infected mice at 7 DPI. **(C)** CD69 expressing $CD4^+$ T cells in infected mice at 21 DPI show significant decrease than infected mice at 7 DPI. **(D)** Absolute number of $Ki67^+$ $CD4^+$ T cells shows non-significant changes. **(E)** The absolute number of $CD8^+$ T cells in infected mice at 7 DPI shows non-significant changes. **(F)** CD69 expressing $CD8^+$ T cells in infected mice at 21 DPI show significant decrease than infected mice at 7 DPI. **(G)** $Ki67^+$ $CD8^+$ T cells in infected mice show non-significant change. **(H)** IFN-γ^+ $CD4^+$ T cells in infected mice at 21 DPI shows significant decrease than controls and infected mice at 7 DPI. **(I)** Absolute number of TNF-α expressing $CD4^+$ T cells in infected mice at 7 DPI shows significant increase than the control and infected mice at 21 DPI. **(J)** Absolute number of IL-17 expressing $CD4^+$ T cells in infected mice at 7 DPI shows significant increase compared to both the control and infected mice at 21 DPI. **(K)** IFN-γ expression by $CD8^+$ T cells in infected mice at 7 DPI shows significant increase than the control and infected mice at 21 DPI. **(L)** TNF-α expressing $CD8^+$ T cells in infected mice at 7 DPI show significant increase than the control. *, **, *** indicate $p<0.05$, <0.01, <0.001 respectively. Data are presented as mean ± SEM of 3-5 mice/group and representative of three independent experiments.

Figure S3

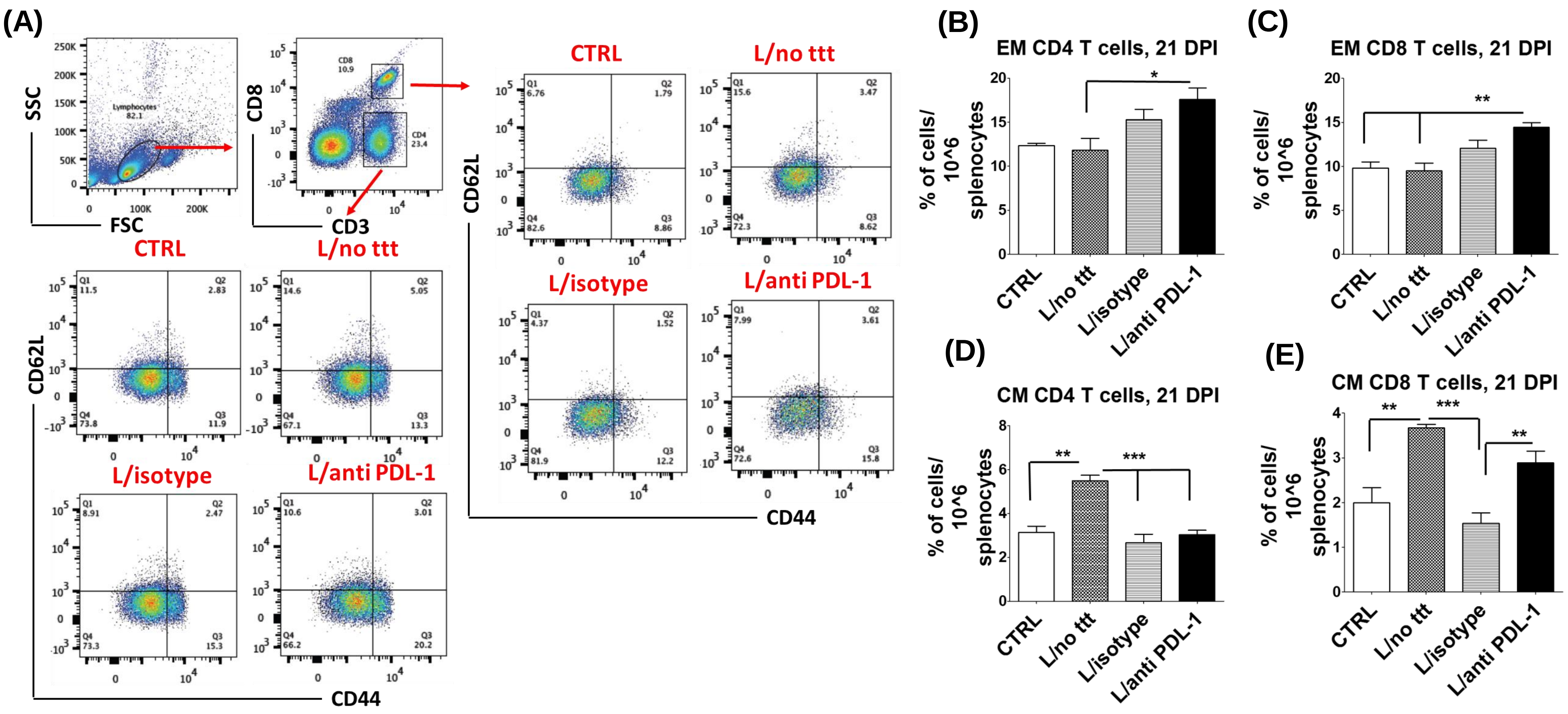

(A)
SSC
FSC
Lymphocytes
82.1
CD8
CD3
CD8
10.9
CD4
23.4
CTRL
L/no ttt
L/isotype
L/anti PDL-1
CD62L
CD44
(B)
EM CD4 T cells, 21 DPI
% of cells/ 10^6 splenocytes
CTRL
L/no ttt
L/isotype
L/anti PDL-1
(C)
EM CD8 T cells, 21 DPI
(D)
CM CD4 T cells, 21 DPI
(E)
CM CD8 T cells, 21 DPI

Fig S3. Anti PDL-1 promotes effector memory $CD4^+$ and $CD8^+$ T cells. Splenocytes were harvested from the following groups of mice: control non-infected, infected non-treated, infected treated with isotype control antibody and infected treated with anti PDL-1 antibody at 21 DPI. Cells were fixed, permeabilized and stained for flow cytometry analysis. **(A)** Gating strategy for effector memory (EM) ($CD44^+$ $CD62L^-$) $CD4^+$ and $CD8^+$ T cells and central memory (CM) ($CD44^+$ $CD62L^+$) $CD4^+$, $CD8^+$ T cells by flow cytometry. **(B)** The percentage of $CD44^+$ $CD62L^-$ (EM) $CD4^+$ T cells in the anti PDL-1 treated group is significantly higher than the infected non-treated group. **(C)** The percentage of $CD44^+$ $CD62L^-$ (EM) $CD8^+$ T cells in the anti PDL-1 treated group is significantly higher than the negative and positive control groups. **(D)** The percentage of $CD44^+$ $CD62L^+$ (CM) $CD4^+$ T cells in the infected non-treated group is significantly higher than all other groups. **(E)** The percentage of $CD44^+$ $CD62L^+$ (CM) $CD8^+$ T cells in the anti PDL-1 treated group is significantly higher than the isotype control group. *, **, *** indicate $p<0.05$, <0.01, <0.001 respectively. Data are presented as mean ± SEM and are from three independent experiments with 4-5 mice/group.

Figure S4

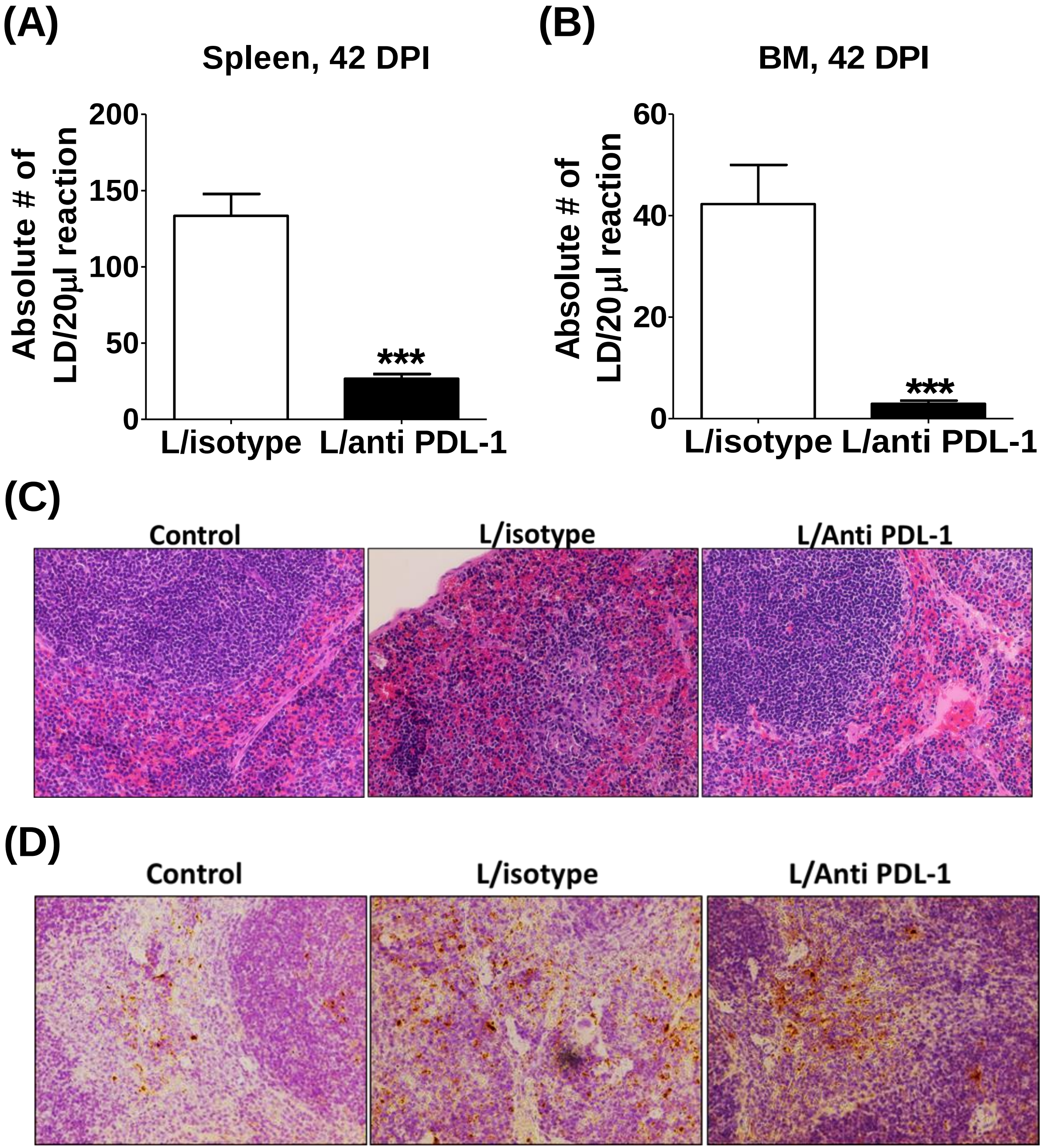
(A)
Spleen, 42 DPI
Absolute # of LD/20μl reaction
200
150
100
50
0
***
L/isotype
L/anti PDL-1
(B)
BM, 42 DPI
Absolute # of LD/20μl reaction
60
40
20
0
***
L/isotype
L/anti PDL-1
(C)
Control
L/isotype
L/Anti PDL-1
(D)
Control
L/isotype
L/Anti PDL-1

Fig S4. Anti PDL-1 continues its anti-leishmanial effect at 42 DPI without causing tissue damage. Anti PDL-1/isotype control was introduced intraperitoneally to infected mice starting at day 7 PI, the indicated mice groups were sacrificed at 42 DPI. **(A)** qRT-PCR for the *L. donovani* gDNA in the spleen and **(B)** bone marrow demonstrating that anti PDL-1 treated mice express highly significant decrease in the parasitic burden compared to the isotype control group. **(C)** H&E stained splenic cuts showing organized architecture of the white pulp in anti PDL-1 treated mice, while the isotype control spleens still show disorganized splenic histology (original magnification 40×). **(D)** IHC for the splenic cuts stained for cleaved caspase 3, showing increased staining in the isotype treated group than both the control and anti PDL-1 treated groups (original magnification 40×). *** indicate $p<0.001$. Data are presented as mean ± SEM of 5 mice/group.
